# Exploration of spontaneous brain activity abnormalities in high myopia patients using resting-state fMRI with multiparameter analysis

**DOI:** 10.3389/fneur.2026.1756996

**Published:** 2026-03-19

**Authors:** Huihui Wang, Hongwei Wen, Qian Chen, ChunMei Cui

**Affiliations:** 1 Department of Radiology, Beijing Chaoyang Hospital, Capital Medical University, Beijing, China; 2 Department of Radiology, Beijing Friendship Hospital, Capital Medical University, Beijing, China; 3 Nuclear Medicine and Radiation Safety Key Laboratory of Sichuan Province, North Sichuan Medical College, Nanchong, China; 4 School of Medical Imaging, North Sichuan Medical College, Nanchong, Sichuan, China; 5 Department of Ophthalmology, Beijing Chaoyang Hospital, Capital Medical University, Beijing, China

**Keywords:** amplitude of low-frequency fluctuation (ALFF), fractional ALFF (fALFF), high myopia (HM), regional homogeneity (ReHo), resting-state functional MRI

## Abstract

**Background:**

High myopia (HM) is a common eye disorder which has become a public health problem globally. Previous neuroimaging studies demonstrated that HM is associated with brain structural abnormalities, whereas the spontaneous brain activity changes in HM are not well studied.

**Methods:**

30 HM patients and 31 healthy controls were included in this study. The altered spontaneous brain activity in HM patients and their relationships with disease durations were explored, using amplitude of low-frequency fluctuation (ALFF), fractional ALFF (fALFF) and regional homogeneity (ReHo) methods based on resting-state functional MRI.

**Results:**

Compared with controls, HM patients showed significant increased ALFF in the left insula, hippocampus, increased fALFF in the right hippocampus, calcarine fissure, superior temporal gyrus, lingual gyrus, increased ReHo in the left middle frontal gyrus, calcarine fissure and right cingulate gyrus, and decreased ALFF, fALFF in the left inferior frontal gyrus (opercular part), left inferior parietal lobule respectively. Besides, the mean ALFF value of left hippocampus and the mean ReHo value of left middle frontal gyrus exhibited significantly positive correlations with disease duration, and the mean fALFF value of left inferior parietal lobule showed significantly negative correlations with disease duration.

**Conclusion:**

HM patients associated with neural dysfunctions in the vision network, attention network and limbic system, which may implicate the presence of neurobiological changes involving deficits in sensorimotor, vision and limbic system in HM patients, providing early useful diagnostic biomarkers for HM as well.

## 1 Introduction

High myopia (HM) is a significant public health challenge worldwide, which characterized by blurred vision. The morbidity rate of HM has reached as high as 2.9% globally, with prevalence rates reaching 10–20% among young adults in East and Southeast Asia (1). By 2050, there will be an estimated 938 million HM individuals (9.8% of the global population) (2). HM is also known as “degenerative myopia” and “pathological myopia,” for the reason that it can induce many fundus complications such as choroidal neovascularization, retinal ischemia, retinal detachment, cataract formation, glaucoma, and potential blindness (3, 4). It exhibits a general trend of degenerative and pathological alterations of the retina and brain structure, may lead to neural activity abnormity and neural plasticity (5–8).

Our previous studies have revealed that HM is accompanied by structural abnormalities, primarily affecting brain regions associated with vision, the default mode network (DMN), sensory/motor modules (9, 10). Apart from these articles on brain structure research of HM, growing functional studies have also exhibited altered neural activities and functional connections in the brain of HM patients (11, 12). These findings confirmed the truth that HM is a progressive neurodegenerative condition impacting the neurosensory retina, brain structure and neural activity. HM is a disorder of both the fundus and the brain, in both structure and function. Thus, probing its associated neural activity alterations is essential to advance our understanding of its pathophysiological basis.

Resting-state functional magnetic resonance imaging (rs-fMRI) provides the optimal visualization of intrinsic neural activity patterns in high myopia (HM). This non-intrusive technique enables researchers to examine spontaneous neural fluctuations through analysis of blood oxygen level-dependent (BOLD) signals. Among various rs-fMRI metrics, amplitude of regional homogeneity (ReHo), low-frequency fluctuation (ALFF)and fractional ALFF (fALFF) are three particularly informative measures. ALFF represent the signal intensity of s spontaneous neural fluctuations through quantifing the total power in the 0.01–0.10 Hz frequency range (13, 14). Moreover, fALFF calculates the ratio of power within low-frequency range to the total power across the entire frequency spectrum, thereby reflecting a normalized measure of low-frequency activity (15). Both ALFF and fALFF excellent measurement consistency across repeated scans, with particularly robust reliability observed in gray matter regions (16). ReHo is a voxel-based measure of temporal BOLD signal similarity (low-frequency, < 0.08 Hz) within a local cluster, thus reflecting the degree of regional neural synchrony (17). By using the ALFF method, Cheng Y, et al. found individuals with myopia and high myopia had abnormal internal brain activities in multiple brain regions related to the DMN, limbic system, and thalamo-occipital pathway (18). Huang et al. (5) showed the HM patients had significantly altered ALFF in brain regions responsible for visual and auditory motion, recognition, language comprehension, motor attention, and so on. However, these two papers utilized only one parameter of ALFF; moreover, the application of the GRF multiple comparison correction method was not sufficiently stringent, applying an excessively lenient voxel-level threshold (*p* < 0.01). It is worth noting that the currently internationally standard for voxel-level significance is defined as *p* < 0.001. In our article, we conducted a comprehensive analysis incorporating the three metrics- ALFF, fALFF and ReHo, using the stringent GRF multiple comparison correction method (voxel level *p* < 0.001, cluster level *p* < 0.05). This approach significantly strengthens the rigor and credibility of the HM analysis. The combined use of these metrics has emerged as a prevalent approach for detecting functional alterations in various brain disorders (19), Alzheimer’s disease (20), Tourette syndrome (21), Cerebral Small Vessel Disease (22) and so on. Accordingly, integrating these three methods will offer more comprehensive assessment of whole-brain intrinsic activity.

Therefore, we aim to not merely investigate the intensity of neural activity through ALFF/fALFF analysis, but also assess neural synchronization using ReHo analysis in HM patients in our article. Additionally, we assessed the clinical associations of these aberrant neural indices. Our findings lay the groundwork for characterizing altered spontaneous neural activity in HM, which may lay the groundwork for advanced understanding and novel therapeutic strategies.

## 2 Materials and methods

### 2.1 Subjects

Our research acquired approval from the ethics committee of our hospital. Before the examination, all participants were required to sign an informed consent. All the procedures involved in this research were carried out in accordance with the principles of the “Helsinki Declaration.” We recruited 35 HM patients from our hospital between January 2017 and December 2020. Additionally, 35 healthy controls (HCs) with uncorrected visual acuity (VA) ≥ 1.0 were enrolled, which were age and gender matched. All patients were instructed to remove any vision-correcting eyewear prior to the examination, and to remain at rest with their eyes closed throughout the procedure. All participants were right-handed. The exclusion criteria encompassed the following: other eye disorders, such as amblyopia, glaucoma, optic neuritis and strabismus; unilateral myopia; psychiatric diseases; systemic Disorder, including diabetes and hypertension; Contraindications to MRI (e.g., cardiac pacemaker, implanted metal devices); MR images with head motion artifacts, brain tumors, infarction of the brain. Based on the above criteria, we five HM patients and four HCs were removed from the study.

### 2.2 Image acquisition

We obtained the MRI data on a 3-Tesla MR scanner (GE Medical Systems, Discovery MR750), which equipped with an eight-channel phased-array head coil. Prior to scanning, participants were instructed to remain awake and maintain quiet, normal breathing throughout the procedure. They were also asked to keep their respiration and heart rate in a stable state to minimize motion artifacts. Functional MRI data were obtained using the following parameters: slice thickness = 3 mm, TR/TE = 2000/24 ms, matrix = 64 × 64, field of view (FOV) = 22 × 22 cm2. For anatomical reference and segmentation, 3D T1WI BRAVO images were obtained with a fast-spin-echo sequence, and the parameters were as follows: slice thickness = 1.0 mm; 200 axial slices; TR/TE = 8.2/3.2 ms; matrix = 512 × 512; FOV = 240 mm × 240 mm.

### 2.3 Data preprocessing

Following data acquisition, 3D T1WI images were manually reoriented to the standard AC-PC coordinate system. Anatomical alignment was performed by defining the anterior commissure (AC), posterior commissure (PC), and mid-sagittal plane, in accordance with standard neuroimaging conventions. Meanwhile, rs-fMRI preprocessing was performed with a customized pipeline based on SPM8^1^ and DPABI 5.1.^2^ To stabilize initial signal fluctuations, the first 10 volumes of functional scans were removed. Subsequent steps included: (1) Slice-timing adjustment to compensate for inter-slice acquisition delays. (2) Head-motion correction via rigid-body transformation, with motion parameters quantified using frame-wise displacement (FD) (23). (3) Exclusion of participants exceeding motion thresholds (max displacement: 3.0 mm/degree; mean FD > 0.2 mm; one healthy subject excluded). (4) Using New Segment module in DPABI, T1WI images were first coregistered to the mean functional image; and were subsequently segmented into gray matter, white matter (WM) and cerebrospinal fluid (CSF). (5) Nuisance regressors, including the Friston-24 head-motion parameters, were removed from the data (24, 25), WM/CSF time-series derived from SPM8 probability masks at 80% tissue probability. (6) Spatial normalization to MNI space via DARTEL (26), combining 12-parameter affine transformation and nonlinear warping, followed by resampling to 3 × 3 × 3 mm^3^ isotropic voxels.

### 2.4 Measurement of ALFF, fALFF, and ReHo

The amplitude-based metrics were computed through spectral analysis of preprocessed BOLD signals. Initially, spatial smoothing was applied to the resampled images with a Gaussian kernel (4 mm FWHM) to enhance signal quality. Spectral power within the 0–0.25 Hz frequency range was computed for each voxel’s time series through a Fast Fourier Transform (FFT). The amplitude of low-frequency fluctuations (ALFF) was derived by calculating the square root of power spectrum values and averaging them within the 0.01–0.08 Hz frequency band. Fractional ALFF (fALFF) was subsequently obtained by normalizing the low-frequency amplitude against the entire spectral power (0–0.25 Hz). Both metrics were standardized by dividing voxel-wise values by the global mean, producing mALFF and mfALFF for statistical analysis.

ReHo was assessed through temporal synchronization analysis of filtered BOLD signals. After spatial normalization, images were bandpass-filtered (0.01–0.08 Hz) to minimize physiological noise interference. The Kendall’s coefficient of concordance (KCC) was computed between each voxel’s time course and its 27 nearest neighbors to quantify local functional coherence (27). F Resulting ReHo values were normalized by the whole-brain mean and spatially smoothed using 4 mm FWHM Gaussian kernel, generating smReHo maps for subsequent statistical comparisons.

### 2.5 Statistical analysis

Initially, a 90% population-based mask (voxels present in ≥90% of participants) was created using DPABI to ensure analysis consistency across subjects. Group differences in whole-brain ALFF, fALFF, and ReHo were compared using the two-sample *t*-test with age, gender, and mean FD included as covariates (23). To further address the potential nonlinear effects of age on fALFF, the quadratic term of age was additionally included as a covariate in a supplementary analysis for fALFF. Multiple comparisons were corrected using the Gaussian random field (GRF) method in DPABI toolbox, with a significance level defined by a combined threshold of *p* < 0.001 at the voxel level and *p* < 0.05 at the cluster level (two-tailed) (28). All neuroanatomical coordinates are reported in Montreal Neurological Institute (MNI) standard space. Clusters demonstrating significant between-group differences were designated as functional regions of interest (ROIs) for subsequent analysis. Then, we extracted the average ALFF/fALFF/ReHo values for each corresponding ROI and performed ROI-based between-group comparisons using two-sample *t*-tests, with age, gender, and mean FD included as covariates. In the patient cohort, mean ALFF/fALFF and ReHo values were extracted from these ROIs for clinical correlation studies. Using SPSS 24.0 (IBM Corp., Armonk, NY), we computed partial correlations between these neural measures and disease duration, controlling for age, sex and FD. The statistical significance level of *p* < 0.05 was applied for all clinical correlations.

## 3 Results

### 3.1 Demographic and clinical characteristics of the subjects

A total of thirty HM patients (11 males, age range of 22–65) and thirty-one HCs (14 males, 24–65 years) were enrolled. The demographic and clinical characteristics of all subjects are summarized in Table 1.

**Table 1:** Demographic and clinical characteristics of high myopia patients and healthy controls.

Characteristic	High myopia patients	Healthy controls	*p*-value
Number of subjects	30	31	
Age (years)	35.13 ± 13.73	38.52 ± 13.43	0.335^a^
Gender (female/male)	19/11	17/14	0.500^b^
FD_Jenkinson	0.082 ± 0.049	0.068 ± 0.047	0.243^a^
Duration of illness (years)	23.8 ± 11.8	NA	
Refractive diopter_R (D)	−8.4 ± 4	NA	
Refractive diopter_L (D)	−8.5 ± 4	NA	
Axial length_R	28.0 ± 1.7	NA	
Axial length_L	27.9 ± 2.2	NA	

NA, not applicable; D, diopter.

a Two-sample *t*-test.

b Chi-square test.

### 3.2 ALFF results

**Figure 1:**
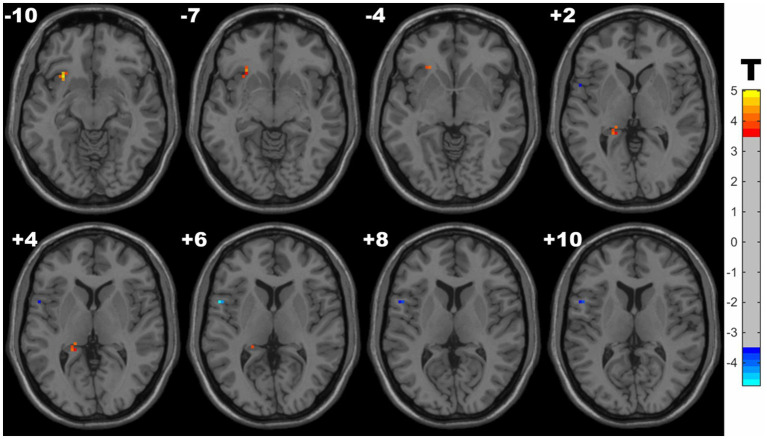
Clusters with significantly increased ALFF value in HM groups (with GRF correction, voxel level *p* < 0.001, cluster level *p* < 0.05). The blue and red areas denote lower and higher ALFF in HM group.

Compared with controls, the HM group exhibited significantly increased ALFF in clusters located in the left insula and hippocampus, and decreased ALFF in the left inferior frontal gyrus (IFG) (opercular part). Detailed results are presented in Table 2 and Figure 1.

**Table 2:** Two-sample *t*-tests demonstrated regions with significantly increased ALFF values in HM patients comparing with controls (with GRF correction, voxel level *p* < 0.001, cluster level *p* < 0.05).

Condition	Brain regions	Cluster size	*z*-score of peak voxel	MNI coordinates of peak voxel		
				*x*	*y*	*z*
HM > HC	Left insula	11	5.04	−30	18	−9
	Left hippocampus	5	4.70	−21	−39	3
HM < HC	Left inferior frontal gyrus, opercular part	5	−4.76	−54	9	6

HM, high myopia; HC, healthy control.

### 3.3 fALFF results

**Figure 2:**
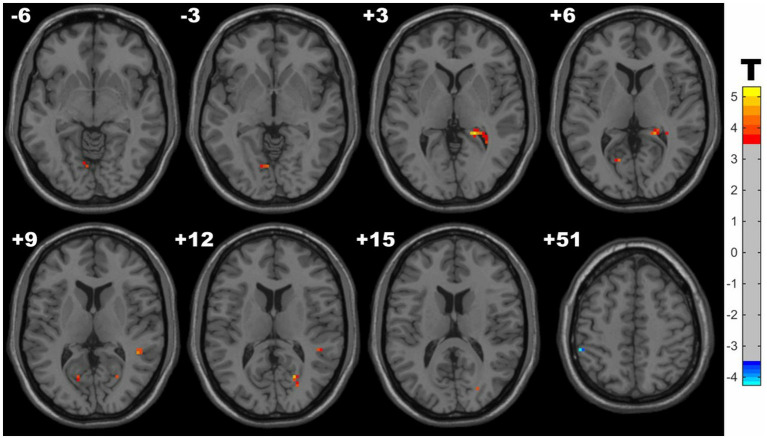
Clusters with significantly altered fALFF value between groups (with GRF correction, voxel level p < 0.001, cluster level *p* < 0.05). The blue and red areas denote lower and higher fALFF in HM group.

Compared with controls, the HM group manifested significantly increased fALFF in the clusters of right hippocampus, calcarine fissure, superior temporal gyrus (STG), lingual gyrus, and decreased fALFF in the left inferior parietal lobule (IPL). Detailed results are presented in Table 3 and Figure 2. Notably, a supplementary analysis controlling for the non-linear effect of age confirmed the robustness of these core findings, with only minor refinements observed (see Supplementary Table S1).

**Table 3:** Two-sample *t-*tests demonstrated regions with significantly altered fALFF values in HM patients comparing with controls (with GRF correction, voxel level *p* < 0.001, cluster level *p* < 0.05).

Condition	Brain regions	Cluster size	*z*-score of peak voxel	MNI coordinates of peak voxel		
				*x*	*y*	*z*
HM > HC	Right hippocampus	18	5.32	21	−39	3
	Right calcarine fissure	7	4.77	21	−66	12
	Right superior temporal gyrus	6	4.47	45	−42	9
	Left lingual gyrus	5	4.25	−6	−72	−3
	Left calcarine fissure	4	4.30	−15	−66	6
HM < HC	Left inferior parietal lobule	4	−4.26	−54	−39	51

### 3.4 ReHo results

**Figure 3:**
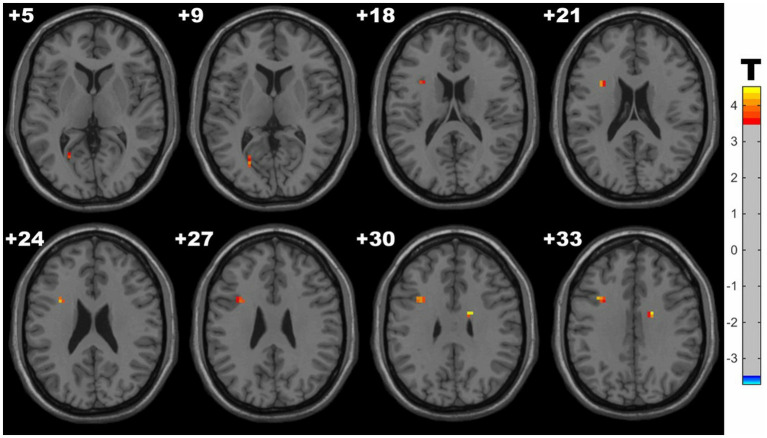
Clusters with significantly increased ReHo value in HM groups (with GRF correction, voxel level *p* < 0.001, cluster level *p* < 0.05). The red-yellow areas denote higher ALFF in HM group.

Compared with controls, HM group showed significantly increased ReHo in the clusters of left middle frontal gyrus (MFG), calcarine fissure and right white matter within cingulate gyrus. Detailed results are presented in Table 4 and Figure 3.

**Table 4:** Two-sample *t*-tests demonstrated regions with significantly increased ReHo values in HM patients comparing with controls (with GRF correction, voxel level *p* < 0.001, cluster level *p* < 0.05).

Condition	Brain regions	Cluster size	*z*-score of peak voxel	MNI coordinates of peak voxel		
				*x*	*y*	*z*
HM > HC	Left middle frontal gyrus	26	4.21	−36	12	33
	Right cingulate gyrus	7	4.52	18	−3	30
	Left calcarine fissure	6	4.11	−24	−69	9

### 3.5 ROI-based comparisons and correlation results

**Figure 4:**
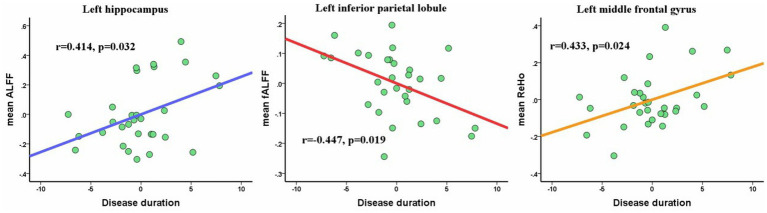
Partial correlations between mean ALFF/fALFF/ReHo value of altered clusters and disease duration. Of note, the coordinate value of both *X*-axis (disease duration) and *Y*-axis (ALFF/fALFF/ReHo value) do not reflect the initial values of these variables, while considering age, gender, and FD as covariates.

The mean ALFF, fALFF, and ReHo values were extracted from significant clusters as ROIs for both groups. After ROI-based between-group comparisons, the results (shown in Supplementary Table S2) demonstrated that the between-group differences in all ROIs remained statistically significant (*p* < 0.05, Bonferroni-corrected), providing additional, independent support for whole-brain findings. In the patient cohort, the mean ALFF, fALFF, and ReHo values were extracted from significant clusters in the HM group for further analysis. Partial correlation analysis indicated that disease duration was positively correlated with the mean ALFF in the left hippocampus (*r* = 0.414, *p* = 0.032) and the mean ReHo in the left MFG (*r* = 0.433, *p* = 0.024). Conversely, the mean fALFF value in the left IPL showed a significant negative correlation with disease duration (*r* = −0.447, *p* = 0.019; Figure 4).

## 4 Discussion

In our study, the combination of ALFF/fALFF and ReHo approaches are used to explore the intrinsic neural activities in HM patients. We found that there were significant differences in the three parameters of ALFF/fALFF and ReHo in HM patients, and the altered areas mainly included visual module, attentional function module and limbic system. These methods are based on different neurophysiological mechanisms, ALFF/fALFF analysis reflects neural intensity, whereas ReHo represents neural coherence (21). The altered neural activities in certain brain regions detected in HM patients in our research contributed to a deeper understanding and exploration of the pathogenesis of this disease.

### 4.1 Major altered parameters and their clinical significance

HM group revealed enhanced fALFF in bilateral calcarine fissure and left lingual gyrus, as well as enhanced ReHo in left calcarine fissure compared to the controls. The calcarine cortex and lingual gyrus, integral components of primary visual cortex (V1), serve as critical anatomical regions that initially receive and process visual information from the visual pathway before relaying these signals to higher-order visual cortical areas for further interpretation and integration (29). The calcarine fissure belongs to the core region of V1. Previous neuroimaging studies have consistently identified structural and functional changes in the visual cortex of patients with ocular disorders. Li et al. (30) identified a higher white matter volume in the calcarine area using voxel-based morphometry analysis. Ji et al. (31). found significant alterations in dynamic functional connectivity between V1 and other brain regions. These findings are in line with previous reports of altered ALFF and ReHo values in the calcarine cortex and lingual gyrus across various ocular conditions, including blindness and retinitis pigmentosa (31). Consistent with these previous findings, our study revealed increased fALFF and ReHo values in the calcarine fissure and lingual gyrus of HM patients. These neurophysiological changes likely reflect enhanced local brain activity, potentially representing a compensatory mechanism and in response to visual system alterations associated with HM.

Decreased ALFF in the left IFG, decreased fALFF in the left IPL and enhanced ReHo in the right MFG in HM individuals were obsevered in our research. These findings are particularly noteworthy as the IFG, IPL and MFG collectively constitute core components of the brain’s attention module, suggesting potential functional modifications in attentional-related processing in HM patients. IFG is a component of the frontal lobe and play a crucial role in language comprehension. Besides, IPL participates in the encoding of spatial positions and plays a key role in spatial attention and motor attention (5). MFG is part of frontal eye field (FEF) and responsible for saccadic and voluntary eye movement (32). Huang et al. (5) showed HM patients had significantly lower ALFF in the bilateral IFG, right MFG, and right IPL and higher ALFF values in the left IPL. Lu et al. (32) exhibited that anisometropic amblyopia children manifested significantly reduced ReHo of spontaneous brain activity in the right MFG. Li et al. (30) demonstrated that HM individuals showed smaller white matter volume in the prefrontal area. While minor discrepancies exist between these previous reports and our current analysis, potentially attributable to the different groups and courses of patients. Consistent with our study, neural activity abnormalities in IFG, IPL and MFG were found in patients with various ocular disorders. Our findings suggested that HM patients have functional impairments in attention module, including language comprehension, spatial or motor attention, eye movement and so on.

The limbic system was also implicated by the aberrant changes in neural activity observed in HM, mainly manifested as elevated ALFF value in left insula and hippocampus, increased fALFF value in right hippocampus and increased ReHo value in the right cingulate gyrus. The hippocampus is located on the medial aspect of the temporal lobe, plays a pivotal role in cognitive functions such as memory and spatial guidance (33). Spatial navigation and the brain’s visual system are closely interconnected, facilitating their functional interaction. The observed enhancement of bilateral hippocampal neural activity in our study indicate that long-term visual impairment may causes compensatory changes in hippocampal neural activity. The insula is a component of the multisensory integration center, plays a pivotal role in ocular movement and spatial orientation (10). The cingulate gyrus orchestrates processing across multiple cognitive domains, such as attention, memory, and executive control (34). In our previous article on the topological properties of white matter research, HM patients were found to be present additional hub regions in the left insula and cingulate (10), which demonstrated long-term myopia leads to the hub-distributing reorganization in these brain regions. Abnormalities in neural activity within the insula (35) and cingulate gyrus (36) have been documented in visual-related diseases. In line with these findings, our study demonstrated that long-term high myopia can impair cognitive function and trigger compensatory neural activity in these brain regions.

Additionally, an increased in the fALFF value was obsevered in the STG. The STG, along with the middle temporal visual area (V5/MT+), constitutes a part of the dorsal visual pathway, which is responsible for processing visuospatial information such as spatial recognition and motion perception (32). Furthermore, the STG contains the primary auditory cortex and is critical for processing auditory information (32). Increased neural activities in the STG were obsevered in amblyopia in previous study (34, 37). The findings in our study may imply a potential compensatory functional and mechanism reorganization in visual–auditory integration in patients with visual disfunction.

### 4.2 Correlations between imaging parameters and clinical variables

The correlations between ALFF, fALFF andReHo values in the identified brain regions and clinical variables were analyzed. Results showed the mean ALFF value in the left hippocampus and the mean ReHo value in the left MFG were positively correlated with disease duration. and the mean fALFF value of left inferior parietal lobule showed significantly negative correlations with disease duration.

The hippocampus plays a crucial role in cognitive functions such as memory and spatial guidance (33), while the IPL and MFG are primarily involved in attention-related processing, including saccadic and voluntary eye movements (32). In our study, the observed hyperfunction of the left hippocampus and MFG—along with their positive correlations—and the hypofunction of the left IPL and its negative correlation with disease duration indicate a gradual decline in cognitive and attentional functions. Concurrently, compensatory mechanisms appear to operate in an effort to preserve and optimize these functions. Meanwhile, these findings also demonstrate that neuroimaging indicators can effectively detect abnormal neural activity and offer valuable insights for monitoring disease progression.

## 5 Limitations

There had several limitations in this article: (1) Lack of data on educational levels and family history of the disease, which may influence the outcomes of our study; (2) Some elderly subjects were recruited in our study. However, we rigorously age-matched the participants across the two groups. Consequently, we are confident that age-related factors had minimal impact on the observed results; (3) The neural activity alterations associated with HM may vary across different age groups, and age-stratified analysis could yield more robust findings. (4) Despite its wide application, the interpretation of ALFF findings should be tempered by an acknowledgment of its potential susceptibility to physiological confounds. Although we have also presented the more robust fALFF metric to provide a more comprehensive perspective, the inherent limitation of ALFF remains. Therefore, future work will involve expanding the sample size and stratifying subjects by age to enable a more detailed investigation.

## 6 Conclusion

By assessing ALFF, fALFF, and ReHo in HM patients, this work provides insights into the altered spontaneous brain activity associated with the condition. The observed disruptions in the visual, attention, and limbic networks lend support to the involvement of these systems in HM’s pathophysiology, clarifying key aspects of its underlying neurophysiological mechanisms. Importantly, these findings suggest that high myopia should not be regarded merely as an ocular disorder, but rather as a “cerebro-visual” condition—a perspective that calls for more integrated neuro-ophthalmic evaluation in clinical management.

## Data Availability

The raw data supporting the conclusions of this article will be made available by the authors, without undue reservation.

## Footnotes

1
https://www.fil.ion.ucl.ac.uk/spm/

2
http://www.restfmri.net
