# Supplementary material for: Exploration of spontaneous brain activity abnormalities in high myopia patients using resting-state fMRI with multiparameter analysis
Source: Front Neurol. 2026 Mar 19;17:1756996. doi: 10.3389/fneur.2026.1756996 (PMC13043415; doi:10.3389/fneur.2026.1756996)
Supplement: Supplementary file 1 [file Data_Sheet_1.pdf]

Supplementary

Supplementary Table S1. Regions with significantly altered fALFF values in HM patients comparing with controls, using age^2, gender and mean FD as covariates.

**Condition**	**Brain regions**	**Cluster size**	**z-score of peak voxel**	**MNI coordinates of peak voxel**		
				x	y	z
**HM>HC**	Right hippocampus	9	4.92	18	-39	3
	Right calcarine fissure	4	4.91	21	-66	12
	Right superior temporal gyrus	5	4.48	45	-42	9
	Left calcarine fissure	4	4.02	-15	-66	6
**HM<HC**	Left inferior parietal lobule	6	-4.25	-54	-39	51
	Right inferior parietal lobule	7	-4.11	-54	-42	-24

Abbreviations: HM, high myopia; HC, healthy control.

Supplementary Table S2. ROI-based comparisons of ALFF, fALFF, and ReHo between groups.

Metric	ROI	HC	HM	T	P_{cor}
ALFF	Left insula	0.712±0.089	0.894±0.170	5.414	<0.0001
	Left hippocampus	0.703±0.139	0.915±0.222	3.952	0.0026
	Left IFGoperc	1.541±0.474	1.185±0.268	3.996	0.0023
fALFF	Right hippocampus	0.821±0.600	0.932±0.069	6.371	<0.0001
	Right CAL	0.921±0.101	1.039±0.100	5.233	<0.0001
	Right STG	0.913±0.101	1.028±0.098	4.669	0.0002
	Left lingualgyrus	0.985±0.092	1.114±0.133	4.436	0.0005
	Left CAL	0.947±0.119	1.079±0.118	4.428	0.0005
	Left IPL	1.209±0.144	1.067±0.117	4.390	0.0006
ReHo	Left MFG	0.830±0.174	0.993±0.164	3.578	0.0087
	Right cingulategyrus	0.619±0.071	0.708±0.100	3.790	0.0044
	Left CAL	0.723±0.123	0.835±0.115	3.087	0.0377

Abbreviations: HM, high myopia; HC, healthy control; IFGoperc, inferior frontal gyrus, opercular part; STG, superior temporal gyrus; CAL, calcarine fissure; IPL, inferior parietal lobule; MFG, middle frontal gyrus; P_{cor}, p value after Bonferroni correction.
